# Outcomes of the Next In Vitro Fertilization Cycle in Women with Polycystic Ovary Syndrome after a Failed In Vitro Maturation Attempt

**DOI:** 10.3390/jcm12175761

**Published:** 2023-09-04

**Authors:** Wei Guo, Yalan Xu, Tian Tian, Shuo Yang, Rong Li, Jie Qiao, Xiaoying Zheng

**Affiliations:** 1 Centre for Reproductive Medicine, Department of Obstetrics and Gynecology, Peking University Third Hospital, No. 49 North Huayuan Road, Haidian District, Beijing 100191, China; guowei20511055@163.com (W.G.); xuyalan0391@163.com (Y.X.); tiantianpku@126.com (T.T.); yangshuo@263.net (S.Y.); roseli001@sina.com (R.L.); 2 National Clinical Research Centre for Obstetrics and Gynecology, Beijing 100191, China; 3 Key Laboratory of Assisted Reproduction, Peking University, Ministry of Education, Beijing 100191, China; 4 Beijing Key Laboratory of Reproductive Endocrinology and Assisted Reproductive Technology, Beijing 100191, China; 5 Research Units of Comprehensive Diagnosis and Treatment of Oocyte Maturation Arrest, Chinese Academy of Medical Sciences, Beijing 100006, China

**Keywords:** in vitro fertilization (IVF), in vitro maturation (IVM), polycystic ovary syndrome (PCOS), subsequent treatment cycle

## Abstract

Background: In vitro maturation (IVM) is indicated in women with polycystic ovary syndrome (PCOS) who have a very good ovarian response during in vitro fertilization (IVF) and are therefore at high risk of ovarian hyperstimulation syndrome (OHSS). According to the latest practice committee document, IVM could be a major advance in assisted reproductive technology (ART) procedures (reduced cost and simplified treatment); nevertheless, retrospective studies of IVM versus IVF still demonstrate lower chances of a live birth with IVM. Could IVM prove to be an optimal first-line treatment approach? And limited information is available concerning the success of the subsequent IVF cycle after the failure of an IVM cycle. Does IVM treatment adversely affect the subsequent IVF cycle, and is this worth considering before performing the IVF cycle for women with PCOS? Methods: This prospective nested case–control study at the Peking University Reproductive Medicine center in China was performed between March 2018 and September 2020. Women aged 20–38 years with PCOS and infertility and who were scheduled for their first IVF attempt were eligible. A total of 351 women were randomly allocated to receive one cycle of unstimulated natural IVM (*n* = 175) or one cycle of standard IVF with a flexible GnRH antagonist protocol followed by hCG as an ovulation trigger (*n* = 176). This study involved 234 women (58 women with no blastocysts in the first IVM cycle and 158 women who underwent the first IVF cycle). Cumulative live birth rate at 12 months after oocyte retrieval and OHSS of a standard controlled ovarian stimulation (COS) IVF cycle were compared between 58 women in an IVF cycle following a failed IVM cycle and 158 women who underwent the first IVF cycle. Results: No significant differences were found in the cumulative live birth rate (CLBR), ongoing pregnancy rate, or clinical pregnancy rate at 12 months after oocyte retrieval between the two groups (56.9% vs. 58.9%, *p* = 0.795; 58.6% vs. 60.8%, *p* = 0.776; and 84.5% vs. 76.0%, *p* = 0.178). The incidence of moderate-to-severe OHSS was not significantly different between the groups (6.9% vs. 5.7%, *p* = 0.742). Additionally, there were no significant differences in the total gonadotropin dose, stimulation duration, number of retrieved oocytes, number of retrieved mature oocytes, or fertilization rates. Conclusions: Even if the first IVM attempt failed in subfertile women with PCOS, comparable cumulative live birth rates were observed in the subsequent IVF cycle. IVM treatment does not adversely affect the subsequent IVF cycle.

## 1. Introduction

Polycystic ovary syndrome (PCOS) occurs in 5–10% of reproductive-aged women, and 50% of women with PCOS present with infertility [1]. According to the ESHRE/ASRM recommendations, in vitro fertilization (IVF) is the third-line treatment for PCOS and is the next step when oral fertility drugs and gonadotrophin have failed [2]. In IVF treatments, women with PCOS undergo ovarian stimulation by gonadotrophin which produces a large number of follicles leading to a high risk of developing ovarian hyperstimulation syndrome (OHSS). OHSS is an iatrogenic adverse complication of ovarian stimulation (COS) [3].

Compared with IVF, in vitro maturation (IVM) is a procedure that involves reduced costs, less time, and simplified treatment for infertile women with PCOS, which results in a reduced psychosocial impact [4,5]. In the absence of ovarian stimulation, IVM is not associated with OHSS [6,7]. In 2021, ASRM submitted a new practice committee paper indicating that IVM is no longer an experimental technique. Although the clinical outcomes in IVM cycles were initially suboptimal, more recently, researchers report that in centers with IVM expertise, the live birth rates have improved to more than 40% [8]. However, retrospective studies of IVM in most general medical centers versus IVF still demonstrate lower rates than the live birth rate [9]. IVM is rarely used as the first fertility treatment for women with PCOS, who are often advised to undergo IVF instead.

Is IVM worth considering before performing the COS-IVF cycle for women with PCOS? Does IVM treatment adversely affect the subsequent IVF cycle? Limited information is available concerning the success of the next IVF cycle after the failure of an IVM cycle. In 2010, Agdi et al. showed that previous IVM treatment was related to an increase in the mature oocytes and embryos in subsequent IVF cycles, although the clinical pregnancy rate and the incidence of moderate and severe OHSS showed no significant differences [10]. Ferraretti et al. reported for the first time that transvaginal ovarian drilling improved IVF outcomes in refractory-to-therapy PCOS women and that the IVF cycle resulted in significantly higher fertilization and embryo cleavage rates after transvaginal ovarian drilling [11]. Furthermore, other studies have demonstrated that IVM, due to its “ovarian drilling” effect, which may be beneficial to IVF treatment, further influences outcomes [12].

In this prospective nested case–control study, we aimed to compare the outcomes of the subsequent IVF cycle following a failed IVM cycle and those who underwent the first IVF cycle, and explore the potential impact of these factors on the risk of clinical pregnancy outcomes, including the general characteristics of both couples, and the IVF laboratory indicators during natural IVM and IVF cycle treatment. Our research data reinforce the knowledge on this topic, which is currently limited by a small amount of research.

## 2. Materials and Methods

### 2.1. Dissemination and Ethics

The research was approved by the Ethics Committee of Peking University Third Hospital (registration number: 2017sz-066). All recruited couples provided informed consent for the procedures, with all information handled confidentially.

### 2.2. Study Design (Figure 1)

**Figure 1:**
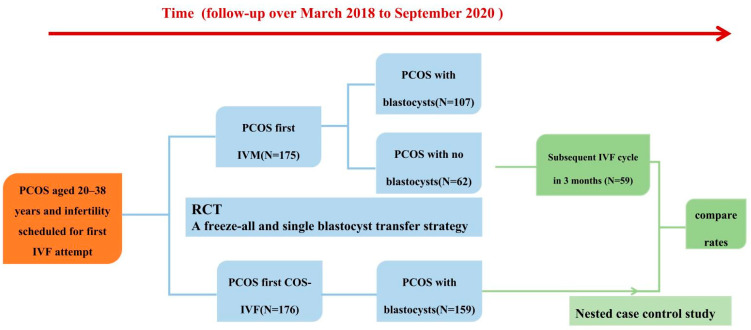
Outcomes of the next IVF cycle in women with PCOS after a failed IVM attempt.

The flow chart of this present research process is depicted in Figure 1.

#### 2.2.1. The Protocol of a Preliminary Study

**Figure 2:**
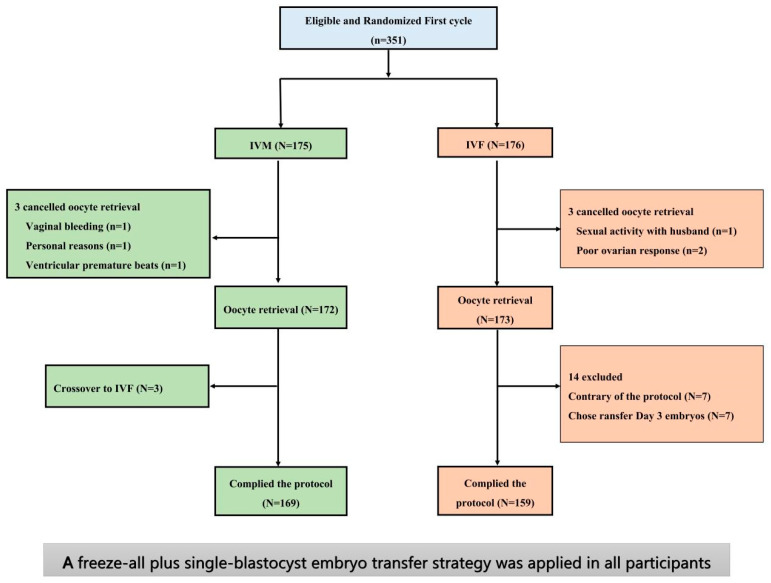
Enrollment, Randomization, and Flow chart in randomized controlled non-inferiority trial in our preliminary study.

We carried out a preliminary study at the Peking University Reproductive Medicine center in China. An open-label randomized controlled non-inferiority trial was performed between March 2018 and July 2019. Women with PCOS and infertility aged 20–38 years and who were scheduled for the first IVF attempt were eligible. A total of 351 women were randomly allocated to receive a cycle of unstimulated natural IVM (*n* = 175) or a cycle of standard IVF with a flexible GnRH antagonist followed by hCG as an ovulation trigger (*n* = 176). Both groups were treated with freeze-all and single-blastocyst transfer strategies. A detailed study protocol and RCT research have been published previously (Figure 2) [6,7].

The inclusion criteria were infertile women with PCOS aged 20–38 years undergoing their first IVF treatment [6,7], and the revised Rotterdam ESHRE/the ASRM present criteria to determine the diagnosis of PCOS [2]. The exclusion criteria were women undergoing fertility preservation and those undergoing the preimplantation genetic testing cycle (PGT).

#### 2.2.2. The Protocol of the Prospective Nested Case–Control Study

**Figure 3:**
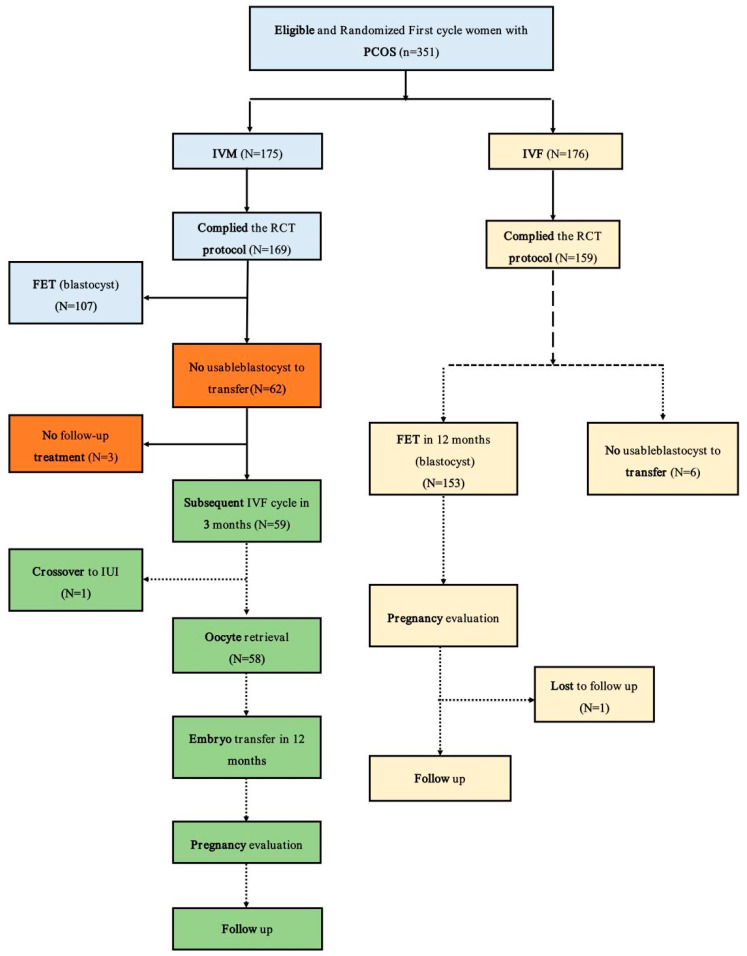
Compare the outcomes of the subsequent IVF cycle following a failed IVM and women with PCOS who underwent the first IVF cycle. COS, controlled ovarian stimulation; IVF, in vitro fertilization; IVM, in vitro maturation; IUI, intrauterine insemination.

This prospective nested case–control study involved 234 women (58 women with no blastocysts in the first IVM cycle and 158 women who underwent the first IVF cycle) from March 2018 to September 2020 at the Center for Reproductive Medicine, Peking University Third Hospital (Figure 1 and Figure 3).

The included patients were infertile women in our preliminary study.

In the study group, 58 women had no blastocysts in the first IVM cycle, and they underwent a subsequent IVF treatment cycle within 3 months.

In the control group, for 158 women who received their first IVF attempt with a flexible GnRH antagonist protocol followed by hCG as ovulation trigger, blastocyst culture was performed for all embryos. All transferable blastocysts were vitrified and then thawed, and a single blastocyst was applied.

### 2.3. Natural IVM Plus Subsequent IVF Cycle (the Study Group)

In the study group, oocytes were retrieved from unstimulated ovaries, and all cumulus–oocyte complexes (COCs) were transferred into the IVM medium. Metaphase II (MII) oocytes were inseminated using intracytoplasmic sperm injection (ICSI), and the embryonic development of cleavage was evaluated according to the developmental stage and the degree of cytoplasmic fragmentation. A good embryo is defined as 5–8 cells with less than 30% fragmentation and an even size. All participants were treated with a total freezing plus single-blastocyst embryo transfer strategy.

After IVM, the women had no blastocysts for transfer and underwent the subsequent IVF treatment cycle.

The use of a GnRH antagonist in conjunction was used for ovarian stimulation. Follicle-stimulating hormone (FSH) (Gonal-F; Serono, Aubonne, Germany) at a starting dose that ranged from 112.5 to 225 IU was started on Days 2–3 of the menstrual cycle. The dose of FSH was adjusted according to the ovarian response, which was monitored by hormone tests and transvaginal ultrasound scanning. The GnRH antagonist (Cetrotide; Serono, Aubonne, Germany), at a dose of 0.25 mg daily, was administered subcutaneously once a lead follicle of 12 mm was observed, up to and including the ovulation trigger day. When 2 or more follicles reached a diameter of at least 17 mm, recombinant hCG 250 mg (Ovidrel; Serono, Aubonne, Germany) or triptorelin 0.2 mg (Diphereline; Ipsen, Beaufour, France) was given for the final ovulation trigger.

### 2.4. Controlled Ovarian Stimulation (COS) First IVF Cycle (the Control Group)

The control group included women who received their first IVF attempt with a flexible GnRH antagonist protocol and hCG as the ovulatory trigger of the flexible GnRH antagonist protocol, and the same protocol described above was followed.

### 2.5. Step-by-Step Descriptions of the IVF Procedures: Oocyte Retrieval, In Vitro Fertilization, and Embryo Culture

Oocyte retrieval was performed 36 (±2) hours after triggering with the use of intravenous sedation; oocytes were inseminated by IVF or ICSI according to the quality of the sperms. In embryo culture, 3 days through the morphological standard evaluation of embryo quality, mainly on the basis of the amount of cleavage ball, regularity, and broken rate.

In the study group, due to a failure of blastocyst culture in the first IVM treatment, the performance of cleavage-stage embryo or blastocyst culture was determined by physicians according to the conditions of the women in the subsequent IVF cycle. In a normal IVF cycle, one to two fresh embryos were transferred, and any remaining suitable embryos were frozen. To reduce the risk of multiple pregnancies, in fresh and frozen embryo transfer cycles, up to two embryos were transferred on Day 3, or one blastocyst was replaced on Day 5.

In the control group, blastocyst culture was performed for all embryos. All transferable blastocysts were vitrified and then thawed and a single blastocyst was applied.

Frozen embryo transfer (FET) was conducted with a hormone replacement therapy (HRT) cycle. The endometrium is administered orally with estradiol valerate (EV) on Day 2 to Day 3 of the menstrual cycle at a dose of 6 mg/day. When the endometrial thickness was ≥7 mm, vaginal progesterone gel (Crinone, Merck Serono) 90 mg/d and oral desdrogesterone 20 mg twice/day were added.

### 2.6. Luteal Phase Supplementation

In the study group, following the fresh transfer, women received 90 mg of vaginal progesterone gel, and 20 mg of dydrogesterone was administered daily for luteal support.

For HRT-FET in both groups, the luteal support was maintained until week 12 of gestation.

### 2.7. Variables and Outcome Assessments

The primary outcome was the cumulative live birth rate within 12 months of oocyte retrieval. Secondary outcomes measures: IVF laboratory and clinical outcomes, maternal medical conditions, obstetric complications, and fetal conditions.

The basal endocrine hormones (luteinizing hormone (LH), follicle-stimulating hormone (FSH), estradiol (E2), progesterone) and AFC were recorded on the 2nd to 3rd day of the menstrual cycle before and after IVM treatment. The total gonadotropin (Gn) dose, stimulation days, number of retrieved oocytes, number of retrieved mature oocytes, fertilization rate, embryo cleavage rate, implantation rate, and number of high-quality embryos were recorded.

Serum β-hCG was detected 12 days after transplantation to confirm pregnancy (β-hCG positive ≥ 10 mIU/mL). Clinical pregnancy was defined as the presence of a gestational sac in the uterine cavity. Ongoing pregnancy was defined as the presence of a viable fetus with a heartbeat at 11 to 12 weeks of gestation. A live birth was defined as a breathing newborn with a heartbeat delivered at ≥28 weeks of gestation. Safety measures included OHSS (classified as mild, moderate, or severe according to the 2016 RCOG guidelines), maternal (miscarriage, ectopic pregnancy) and obstetric and perinatal complications.

### 2.8. Statistical Analysis

Primary hypothesis was that the cumulative live birth rate within 12 months of oocyte retrieval in the IVM-subsequent IVF group would be no significant difference from that in the first IVF group. Continuous variables when normally distributed are presented as means (standard deviations, SDs); in the case of non-normality, medians and inter-quantile ranges (IQRs) are reported. Categorical variables are presented as proportions in each group. For outcome variables, a comparison between groups was performed using the independent sample *t*-test, or medians are compared with the Mann–Whitney U test, Pearson chi-square test, or Fisher’s exact test for categorical variables as appropriate. Statistical analyses were performed using SPSS version 26.0 (Released 2019, IBM Corp., Armonk, NY, USA). The assessment was carried out in a descriptive and correlational manner to address associations between the time interval of oocyte retrieval in the IVM-IVF cycle and different outcomes. All *p* values reported for double tail, with a *p* < 0.05 level of significance.

## 3. Results

### 3.1. Participants

In total, 216 women underwent the research, 58 women complied with the study group protocol (natural IVM plus subsequent IVF cycle), and 176 women with PCOS undergoing their first IVF cycle were selected in the control group; 159 patients complied with the protocol, and 1 patient was lost to follow-up after 12 weeks of gestation and excluded from the outcome analysis (Figure 3).

### 3.2. Demographic and Clinical Characteristics

The demographic and clinical characteristics of the 216 women are shown in Table 1. The median age of women was 29 (27–31) in the study group vs. 29 (27–31.25) in the control group (*p* = 0.574), and 91.40% of women were younger than 35 years in the study group vs. 94.30% in women younger than 35 years in the control group (*p* = 0.574). In 24.10% of infertile couples in the study group, anovulation was the only cause (vs. 17.70% in the control group); 31% of infertile couples had infertility due to anovulation combined with the female reproductive issue (vs. 32.30% in the control group). Additionally, 34.50% had infertility due to anovulation combined with the male reproductive issue (vs. 44.30% in the control group). Finally, 10.30% had infertility due to male and female reproductive problems (vs. 5.70% in the control group) (*p* = 0.185). There were no significant differences between the two groups at the baseline, including a median body mass index (BMI), the duration of infertility and proportion of primary infertility, the number of antral follicles and Day-3 FSH and estradiol, and Anti-Mullerian hormone (AMH).

The interval between the first IVM cycle and the subsequent IVF cycle ranged from 5 days to 80 days (30.98 ± 19.43) in the study group. Basal endocrine hormone levels (basal serum FSH, LH, estradiol, and progesterone levels) on Days 2–3 of the menstrual cycle in the study group before and after IVM treatment are presented in Table 2, and no significant differences were observed.

### 3.3. Primary and Pregnancy Outcomes

In the study group, 4 women had no usable embryos or blastocysts in the subsequent IVF cycle, and 54 women had at least one transfer cycle (Day 3/Day 5). In the control group, 6 women had failed embryo development resulting in no blastocysts for transfer, and 152 women had at least one blastocyst transfer cycle.

Table 3 details the fertility and pregnancy outcomes from all fresh and frozen transfer cycles within 12 months. In the study group, there were 17 fresh and 114 frozen transfer cycles; 85.50% (112/131) of the transferred cycles involving Day-3 embryos (total of 221 cleavage embryos), and 14.50% (19/131) of the cycles involved Day-5 blastocysts (total of 20 blastocyst embryos). In the control group, 152 women (100%) had a single-blastocyst transfer cycle with a total of 244 FET cycles. The cumulative live birth rate, cumulative ongoing pregnancy rate, and clinical pregnancy rate in the study group were similar to those in the control group (56.90% vs. 58.86%, *p* = 0.795; 58.62% vs. 60.76%, *p* = 0.776; and 84.48% vs. 75.95%, *p* = 0.178). The rates of conception, blastocyst implantation, and pregnancy loss were also not significantly different (87.93% vs. 81.01%, *p* = 0.232; 35.00% vs. 49.18%, *p* = 0.249; and 32.65% vs. 25.00, *p* = 0.310).

### 3.4. Treatment and Laboratory Measures

Differences between the two groups regarding the baseline hormone levels characteristics were not significant, including basal serum LH levels between the two groups (6.70 vs. 5.43, *p* = 0.057). The FSH starting dose was higher in the study group (150 IU vs. 150 IU. *p* = 0.00); however, the total FSH dose (*p* = 0.722) and duration of the stimulation treatment (*p* = 0.206) were similar (Table 4). Thus, in the IVM-IVF cycle, a higher level of E2, LH, and *p* were noted compared with those in the IVF cycle (14,428.5 pmol/L in IVM/F cycle vs. 12,441 pmol/L in IVF cycle, *p* = 0.202; 3.43 vs. 2.08, *p* = 0.023; 2.22 vs. 1.96, *p* = 0.027). There were statistically significant differences in serum LH levels on hCG trigger day between the two groups, also serum progesterone levels (*p <* 0.05). The trigger day LH/basal LH (hLH/bLH) was 0.51 vs. 0.38. The mean number of retrieved oocytes was comparable between groups (22.0 vs. 18.0, *p* = 0.959). Overall, 15 cycles (the study group) and 36 cycles (the control group) required ICSI in view of poor semen quality, and the proportion of oocytes that were mature at the time of collection was 78.19% (251/321) and 73.59% (535/727), respectively (*p* = 0.702). The mean number of 2PN and good-quality embryos was comparable (11.0 vs. 10.0, *p* = 0.663; 7.0 vs. 8.0, *p* = 0.066). The incidence of OHSS was similar between the groups: nine women (5.7%) had moderate–severe OHSS in the control group, and four women developed moderate–severe OHSS (6.9%) in the study group (*p* = 0.742); however, almost all patients had symptoms of mild-to-moderate severity.

### 3.5. Effect of the Time Interval between IVM and the Subsequent IVF and Clinical Outcomes

Table 5 shows the correlation between the time interval of oocyte retrieval in the IVM-IVF procedure and different outcomes, a significantly positive correlation with FSH duration of stimulation days (r = 0.321, *p* = 0.014) and the total FSH dose (r = 0.411, *p* = 0.001). However, no significant correlations were observed between the time interval of oocyte retrieval in the IVM-IVF procedure and other clinical outcomes.

## 4. Discussion

The new focus of this study was the effect of ovarian puncture for IVM on subsequent IVF cycles in women with PCOS. Remarkably, the cumulative live birth rate (56.90%) and ongoing pregnancy rate (58.62%) of subsequent IVF treatments were highly favorable and could be achieved in women who were associated with poor outcomes in previous IVM attempts. A limited number of studies on the subject reinforce previous knowledge in the field.

We found that compared with the first conventional IVF cycles, no significant difference in the cumulative live birth rate, ongoing pregnancy rate, or pregnancy outcomes was found in the subsequent IVF cycles performed after an unsuccessful IVM cycle. In 2016, Lin et al. described that previous IVM cycles in women with PCOS resulted in improved endocrine status and increased clinical pregnancy rates during the subsequent IVF cycles [13]. In 2019, Sanne et al. reported that the ongoing pregnancy rate of the first IVF cycle performed after one unsuccessful IVM cycle was 44%, which agreed with our results [14]. Notably, in our study group, women who had no blastocysts in the previous IVM cycle proceeded to a subsequent IVF cycle, and 85.5% of the transferred cycles were in the cleavage stage. The high rates of conception and clinical pregnancy in the control group could be explained by women with transfers in the blastocyst stage having better pregnancy outcomes, while blastocyst embryos have higher implantation rates.

As expected, subsequent IVF laboratory outcomes were improved after IVM in PCOS patients, which means that these punctures may have improved the endocrine characteristics of the patients with PCOS [15]. Compared to the control group, the number of retrieved oocytes, rates of maturation, and fertilization increased in subsequent IVF cycles, but these differences were not statistically significant. No significant between-group differences were observed in high-quality embryos. In 2010, Agdi reported that the application of IVM ovarian puncture increased the number of mature oocytes and embryos in subsequent IVF cycles [10] and suggested that IVM with multiple ovarian punctures to extract immature oocytes may improve the IVF response similarly to LOD. A number of studies have evaluated transvaginal ovarian drilling (TOD) in severe PCOS patients prior to IVF, and a significant improvement was shown in the implantation and pregnancy rates, as well as in the IVF laboratory outcomes (the number of mature oocytes, embryos, and blastocysts) [16,17]. There was no significant difference in male infertility factors; additionally, no statistical differences in fertilization methods (ICSI and IVF) were identified between the two groups. In our center, ICSI offers high fertilization rates and allows the use of semen samples with few sperm or low sperm quality, and in special cases, severe male factor infertility with previous fertilization failure is included in the standard procedure [18,19,20].

Studies have reported that ovarian puncture for IVM significantly decreased serum levels of AMH, luteinizing hormone (LH), and testosterone 5 days after oocyte retrieval and returned to baseline values within 3 months. This procedure resulted in the promotion of ovulation and an increased response to ovarian stimulation, leading to pregnancy in subsequent COS cycles, and it avoided the occurrence of OHSS in women with PCOS [12,21,22]. The time interval between IVM and the subsequent IVF cycle was 30.98 ± 19.43 days (ranging from 5 to 80 days) in our study. For women in whom the first IVM cycle failed, progesterone (dydrogesterone, Duphaston, Abbott, 10 mg twice daily) was used to induce menstruation for the subsequent cycle, and the baseline LH level was mildly elevated on Days 2–3 in the subsequent IVF cycle, which could possibly be due to the use of oral contraceptives for pretreatment in the first IVM cycle. Agdi et al. conducted a study in which the IVM and IVF cycle for the first time interval was 4.1 ± 0.3 months [10]. Unfortunately, they did not have data on hormonal levels before and after IVM treatment.

Ovarian responses to COS vary widely among women with POCS, with some patients having a poor response to COS and others having a high response to COS. In our research, only one patient with very few mature follicles underwent IUI following the subsequent IVF treatments, which suggested that the previous IVM cycle had a good impact on the ovarian response in PCOS. Through correlation analysis, the time interval was positively correlated with the duration of stimulation and total Gn dose.

For women in the IVM group who did not have transferable blastocysts, it was feasible for the woman to restart the next IVF cycle immediately because no stimulating drugs were used by natural IVM. In recent studies, several improvements of IVM have been reported, which had comparable outcomes, but IVM has gained inferiority compared with IVF cycles in terms of clinical pregnancy rate, ongoing pregnancy rate, and live birth rate [4,7,23,24]. Remarkably, our data suggest a potentially beneficial effect for IVM when combined with a subsequent IVF approach, but to investigate the effect of IVM on ovarian reserve, more variables need to be included, including the levels of AMH, inhibin-B, and testosterone. In addition, prospective randomized studies with larger sample sizes are needed.

Another, IVM possibly alter the microenvironment of oocytes could have genetic imprinting abnormalities that are not obvious at present, and the safety issues of IVMs need to be dissected and linked to epigenetic mechanisms or molecular evidence to solidify theories and results, especially the following aspects of different IVM culture methods [25]. Fortunately, the available evidence suggests that newborns born with IVM surgery do not have any problems compared to IVF alone. Our studies over the past decade have not shown an increase in neonatal complications in the IVM group, and further real-world follow-up studies are needed to provide evidence of the long-term safety of IVM [26].

Therefore, the aim of this review is to share our view of the clinical applications of IVM technology. In the absence of an absolute indication for IVF, women with PCOS and anovulatory infertility could be offered IVM as the first-line treatment, which is worth considering before performing IVF and does not adversely affect the outcome of the subsequent IVF cycle.

## 5. Conclusions

Our results reinforce previous knowledge in the field, following on from a limited number of studies on the subject that estimated the potential for IVM with subsequent IVF treatment. Given its good tolerability, few adverse effects, and low cost, IVM treatment could be offered to women with PCOS and is worth considering trying before undertaking an IVF cycle. Counseling on IVM/IVF should be tailored to the specific characteristics of the individual woman. Even if IVM fails, the embryological and clinical outcomes of subsequent IVF treatments were comparable and highly favorable.

## Figures and Tables

**Table 1:** Baseline demographic and clinical characteristics of the participants *.

	IVM-IVF Group (N = 58)	IVF Group (N = 158)	*p* Value
Age (Median [IQR])	29 (27–31)	29 (27–31.25)	0.574
Distribution-no. (%)			0.439
<35 years	53 (91.40%)	149 (94.30%)	
≥35 years	5 (8.60%)	9 (5.70%)	
Body-mass index ^†^—kg/m^2^ (Median [IQR])	24.75 (21.58–27.5)	24 (21.25–27.15)	0.554
Fertility history			
Duration of the attempt to conceive—years (Median [IQR])	3.0 (2.0–5.0)	3.0 (2.0–4.0)	0.377
Previous conception—no. (%)			0.157
0	40 (69.00%)	119 (75.30%)	
1	16 (27.60%)	27 (17.10%)	
≥2	2 (3.40%)	12 (7.60%)	
Indications for IVF—no. (%)			0.185
Anovulation only	14 (24.10%)	22 (17.70%)	
Anovulation combined with other factors			
Female factors	18 (31.00%)	51 (32.30%)	
Male factors	20 (34.50%)	70 (44.30%)	
Both factors	6 (10.30%)	9 (5.70%)	
Ultrasonographic findings			
Antral follicle count in each ovary—no. (%)			0.99
12~20	43 (74.10%)	117 (74.10%)	
>20	15 (25.90%)	41 (25.90%)	
Endometrial thickness—mm (Median [IQR])	5 (5–6.10)	5 (5–6)	0.978
Laboratory tests			
FSH—IU/L (Median [IQR])	6.06 (4.63–7.28)	6.12 (4.69–7.26)	0.997
LH—IU/L ^‡^ (Median [IQR])	7.62 (4.80–12.25)	7.08 (4.40–11.10)	0.364
Estradiol—pmol/L (Median [IQR])	184.00 (127–252.5)	189 (148.95–232.5)	0.53
Hyperandrogenism ^§^ (Distribution-no. (%))	28 (48.30%)	64 (40.50%)	0.306
AMH—IU/L ** (Median [IQR])	9.16 (5.91–15.30)	9.42 (6.26–13.89)	0.778
Number of ovulation induction cycles before randomization (Median [IQR])	2 (0–4)	1 (0–3)	
PCOS phenotype ^#^			0.174
A—no. (%)	17 (29.30%)	45 (28.40%)	
C—no. (%)	13 (22.40%)	20 (12.70%)	
D—no. (%)	28 (48.30%)	93 (58.90%)	

* If the denominators of the patients included in each analysis differ from the totals of the relevant study groups, their denominators are provided. Percentage totals may not reach 100 due to rounding. SD is the standard deviation, and IQR is the interquartile distance. There was no significant difference between the two groups. ^†^ The body-mass index is the weight in kilograms divided by the square of the height in METRE. ^‡^ Luteinizing hormone and follicle-stimulating hormone were measured in units per LITRE. ^§^ Hyperandrogenism is defined as total testosterone > 2.53 nmol/L or androstendione > 11.5 nmol/L. Data of 1 patient (0.55%) in the IVF group were missing. ** Antimullerian hormone data were missing in 9 patients (15.5%) in the IVM group and 11 patients (7.0%) in the IVF group. ^#^ Polycystic ovary syndrome is characterized by clinical and/or biochemical hyperandrogenism (HA), ovulation dysfunction (OD), and polycystic ovary morphology (PCOM). The current phenotypes of PCOS are divided into phenotype a (HA + OD + PCOM), phenotype b (HA + OD), phenotype c (HA + PCOM), and phenotype d (OD + PCOM).

**Table 2:** Baseline hormonal data before and after IVM treatment.

Baseline Characteristics in the Study Group before and after IVM Cycles			
	Before IVM (N = 58)	After IVM (N = 58)	*p* Value
FSH—IU/L (Median [IQR])	5.91 (5.00–7.01)	6.05 (5.07–6.99)	0.821
LH—IU/L (Median [IQR])	5.59 (3.95–10.75)	6.70 (4.47–10.18)	0.639
Estradiol—pmol/L (Median [IQR])	159.0 (126.50–222.00)	167.50 (115.75–213.50)	0.634
Progesterone—nmol/L (Median [IQR])	0.86 (0.64–1.05)	0.93 (0.64–1.29)	0.318

**Table 3:** Cumulative fertility outcomes of women with PCOS within 12 months of the IVF oocyte retrieval.

	IVM-IVF (N = 58)	IVF (N = 158)	Rate Difference (95% CI)	Rate Ratio in the IVM-IVF Group (95% CI)	*p* Value
Total transfer cycles no./total no. (%)					
Cleavage embryos cycle	112 (85.50)				
Blastocyst cycle	19 (14.50)	244 (100)			
Primary outcome					
Live birth—no. (%) ‖	33 (56.90)	93 (58.86)	−0.0196 (−0.1228–0.1672)	0.923 (0.502–1.696)	0.795
Secondary pregnancy outcomes					
Conception—no. (%) ^†^	51 (87.93)	128 (81.01)	−0.0692 (−0.0514–0.1607)	1.708 (0.705–4.135)	0.232
Clinical pregnancy—no. (%) ^‡^	49 (84.48)	120 (75.95)	−0.8448 (−0.0436–0.1869)	1.724 (0.775–3.833)	0.178
Singleton	43	120			
Twins *	6	0			
(Intrauterine with ectopic pregnancy)	1	0			
Implantation (per embryo)—no./total no. (%) ^§^					
D3 cleavage embryo	50/221 (22.62)	0			
D5 blastocyst embryo	7/20 (35.00)	120/244 (49.18)	−0.1339 (−0.0918–0.3137)	0.574 (0.222–1.488)	0.249
Ongoing pregnancy—no. (%) ^¶^	34/58 (58.62)	96/158 (60.76)	−0.0214 (−0.1196–0.1688)	0.915 (0.496–1.688)	0.776
Pregnancy complication					
Pregnancy loss—no./total no. (%)	16/49 (32.65)	30/120 (25.0)	0.0765 (−0.0657–0.2323)	1.455 (0.704–3.007)	0.310
First trimester	14/49 (28.57)	24/120 (20.0)			
Second trimester	2/49 (4.08)	6/120 (5)			

‖ A live birth was defined as a breathing newborn with a heartbeat delivered at ≥28 weeks of gestation. ^†^ Conception was defined as serum hCG ≥ 10 mIU/mL. ^‡^ Clinical pregnancy was defined as the presence of a gestational sac in the uterine cavity. * All twins were derived from patients who requested two cleavage embryo transfer. ^§^ The implantation rate defined as the number of embryos transferred divided by number of gestational sacs on ultrasound. ^¶^ Ongoing pregnancy was defined as the presence of a viable fetus with a heartbeat at 11 to 12 weeks of gestation.

**Table 4:** Oocyte retrieval characteristics and safety secondary endpoints, embryology outcomes of IVM-IVF versus IVF treatment.

	IVM-IVF (N = 58)	IVF (N = 158)	Between Group Difference (95% CI)	*p* Value
Baseline characteristics				
FSH—IU/L (Median [IQR])	6.05 (5.07–6.99)	6.02 (4.84–7.12)		0.796
LH—IU/L (Median [IQR])	6.70 (4.47–10.18)	5.43 (3.95–7.86)		0.057
Estradiol—pmol/L (Median [IQR])	167.50 (115.75–213.50)	167.50 (125.25–218)		0.569
Progesterone—nmol/L (Median [IQR])	0.93 (0.64–1.29)	0.89 (0.64–1.23)		0.768
Oocyte retrieval characteristics				
Duration of stimulation—days	10.0 (9.0–11.0)	10.0 (9.0–12.0)	0.206 (0.194–0.210)	0.206
GN starting dose (IU)	150.0 (150.0–150.0)	150.0 (125.0–150.0)	0.00 (0.00–0.00)	0
GN total dose—IU	1500.00 (1275.00–2034.38)	1500 (1275.00–2296.88)	0.721 (0.713–0.730)	0.722
Estradiol level on hCG trigger day	14,428.5 (7246.25–22,516.00)	12,441 (6920.5–19,503.25)	0.204 (0.196–0.212)	0.202
LH level on hCG trigger day	3.43 (1.11–6.50)	2.08 (1.34–3.58)	0.028 (0.024–0.031)	0.023
*p* level on hCG trigger day	2.22 (1.64–3.20)	1.96 (1.37–2.82)	0.028 (0.024–0.031)	0.027
Trigger day LH/basal LH (hLH/bLH)	0.51 (3.43/6.70)	0.38 (2.08/5.43)		
Safety endpoints—no. total no. (%)				
Moderate–severe OHSS	4/58 (6.9%)	9/158 (5.7%)	1.226 (0.363–4.147)	0.742
Embryology outcomes				
Fertilization method ^‡^				0.233
With IVF	41 (70.7%)	121 (76.6%)		
With ICSI	15 (25.9%)	36 (22.8%)		
With mixed IVF and ICSI	2 (3.4%)	1 (0.6%)		
No. of oocytes retrieved				
Total	1315	3109		
Per patient (median (IQR))	22 (13–29.25)	18 (11.00–25.00)		0.959
No. of mature oocytes				
Total (%)	251/321 (78.19%)	535/727 (73.59%)		
Per patient (median (IQR))	13 (9–23)	13 (8.25, 20)		0.702
No. of 2PN (pronuclear)				
Total (%)	757/1315 (57.57)	1857/3109 (59.7)		
Per patient (median (IQR))	11 (6.75–20)	10 (6, 15)	0.551 (0.541–0.561)	0.663
No. of good—quality embryo				
Total (% per 2PN)	456/757 (60.24)	1390/1857 (74.9)		
Per patient (median (IQR))	7 (3–11.25)	8 (5, 12)		0.066

^‡^ IVF denoted in vitro fertilization. ICSI denoted intracytoplasmic sperm injection. Mixed IVF and ICSI was performed as half ICSI (50% oocytes were inseminated by IVF and 50% oocytes by ICSI).

**Table 5:** The correlation between time interval of oocyte retrieval in IVM-subsequent IVF procedure and different outcomes.

		GN Starting Dose	Duration of Stimulation	GN Total Dose	No. of Oocytes Retrieved	No. of Fertilization	No. of 2PN (Pronuclear) Zygotes	No. of Cleavage	No. of Good—Quality Embryo	No. of Embryos Transferable	No. of Vitrified Blastocysts	Clinical Pregnancy Rate
Time interval of oocyte retrieval in IVM-subsequent IVF (days)	Correlation coefficient	0.072	0.321 *	0.411 **	−0.136	−0.028	−0.017	−0.023	−0.011	−0.045	0.258	0.111
	Significance	0.591	0.014	0.001	0.309	0.836	0.896	0.865	0.934	0.740	0.050	0.409
	N	58	58	58	58	58	58	58	58	58	58	58

**, *p* ≤ 0.001; *, *p* ≤ 0.01.

## Data Availability

The data used to support the findings of this study are available from the corresponding author upon request.
